# Supplementary material for: A new epigenetic framework for DNA methylation, hydroxymethylation, and gene expression during brain aging
Source: bioRxiv. 2026 Oct 21:2026.10.15.757659. Preprint. [Version 1] doi: 10.64898/2026.10.15.757659 (PMC13738379; doi:10.64898/2026.10.15.757659)
Supplement: Supplementary file 1 [file media-1.pdf]

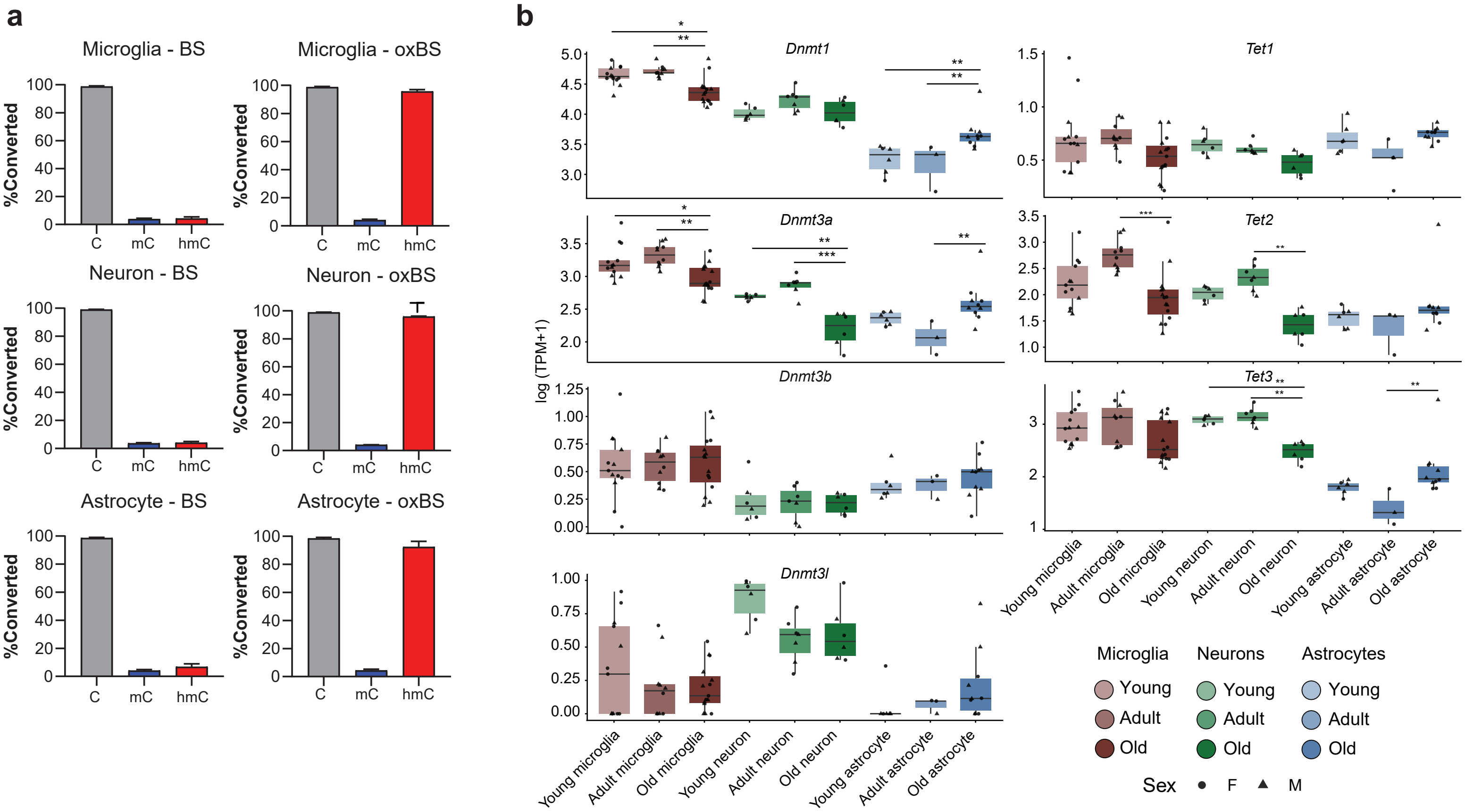
a
Microglia - BS
Microglia - oxBS
Neuron - BS
Neuron - oxBS
Astrocyte - BS
Astrocyte - oxBS
%Converted
C
mC
hmC
b
Dnmt1
Tet1
Dnmt3a
Tet2
Dnmt3b
Tet3
Dnmt3l
log (TPM+1)
Young microglia
Adult microglia
Old microglia
Young neuron
Adult neuron
Old neuron
Young astrocyte
Adult astrocyte
Old astrocyte
Microglia
Neurons
Astrocytes
Young
Adult
Old
Sex
F
M
