# Supplementary material for: A new epigenetic framework for DNA methylation, hydroxymethylation, and gene expression during brain aging
Source: bioRxiv. 2026 Oct 21:2026.10.15.757659. Preprint. [Version 1] doi: 10.64898/2026.10.15.757659 (PMC13738379; doi:10.64898/2026.10.15.757659)
Supplement: Supplementary file 2 [file media-2.pdf]

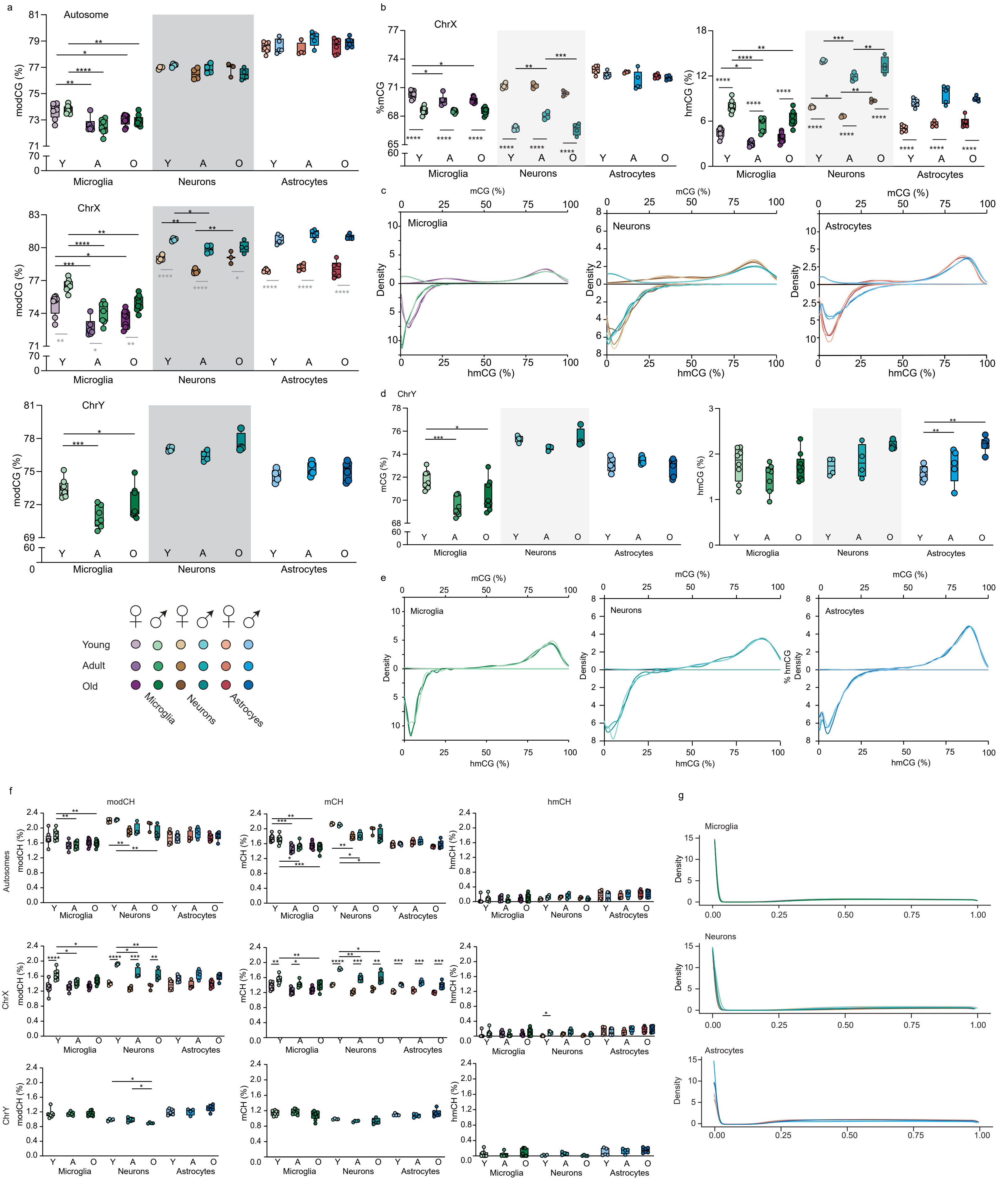

a
Autosome
ChrX
ChrY
modCG (%)
Y A O
Microglia
Neurons
Astrocytes
Young
Adult
Old
b
ChrX
%mCG
hmCG (%)
c
mCG (%)
hmCG (%)
Density
Microglia
Neurons
Astrocytes
d
ChrY
mCG (%)
hmCG (%)
e
f
modCH
mCH
hmCH
Autosomes
ChrX
ChrY
modCH (%)
mCH (%)
hmCH (%)
g
