# Supplementary material for: A new epigenetic framework for DNA methylation, hydroxymethylation, and gene expression during brain aging
Source: bioRxiv. 2026 Oct 21:2026.10.15.757659. Preprint. [Version 1] doi: 10.64898/2026.10.15.757659 (PMC13738379; doi:10.64898/2026.10.15.757659)
Supplement: Supplementary file 3 [file media-3.pdf]

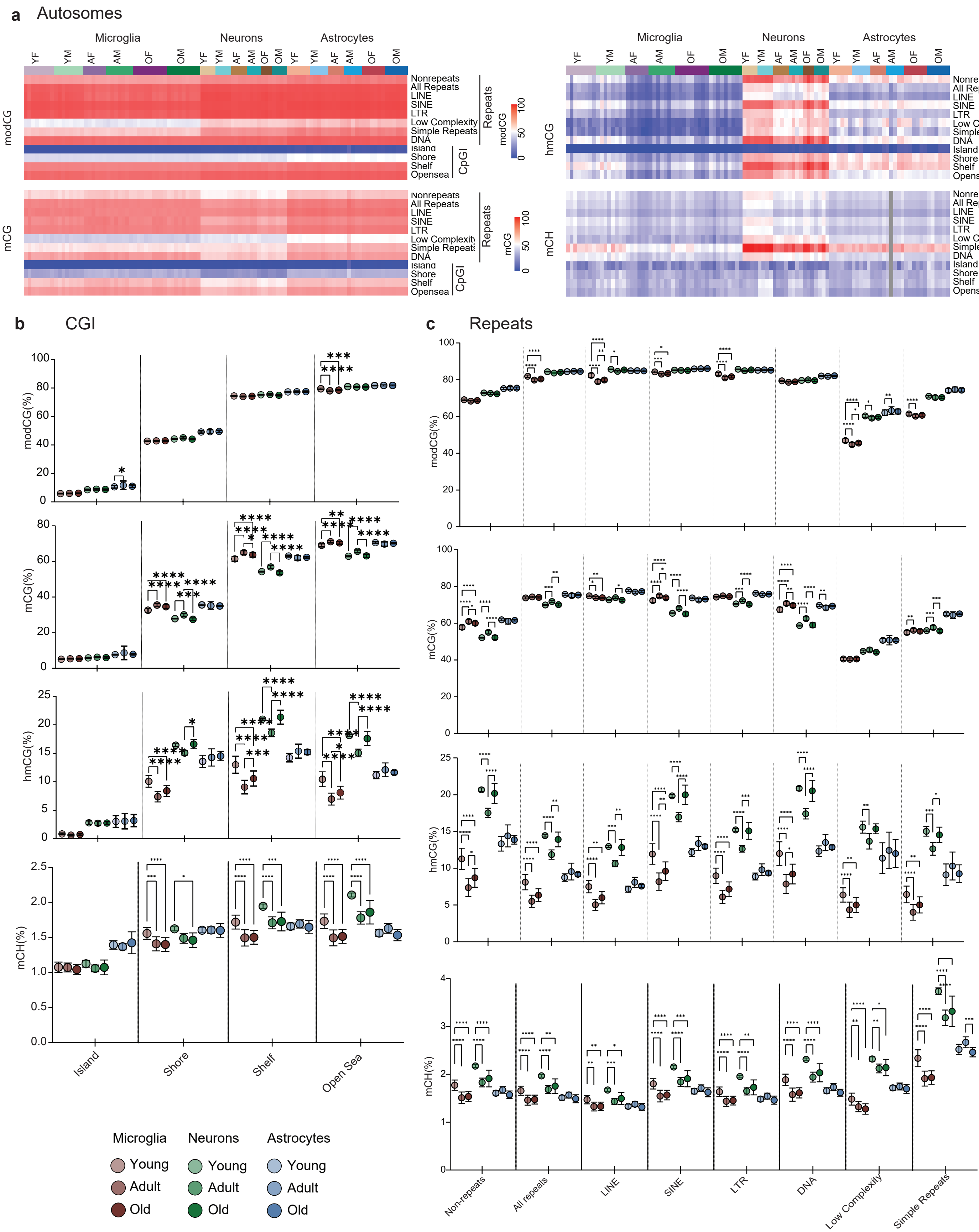
a
Autosomes
Microglia
Neurons
Astrocytes
YF
YM
AF
AM
OF
OM
modCG
mCG
hmCG
mCH
Nonrepeats
All Repeats
LINE
SINE
LTR
Low Complexity
Simple Repeats
DNA
Island
Shore
Shelf
Opensea
Repeats
CpGI
b
CGI
modCG(%)
mCG(%)
hmCG(%)
mCH(%)
Island
Shore
Shelf
Open Sea
c
Repeats
Non-repeats
All repeats
LINE
SINE
LTR
DNA
Low Complexity
Simple Repeats
Microglia
Neurons
Astrocytes
Young
Adult
Old
