# Supplementary material for: A new epigenetic framework for DNA methylation, hydroxymethylation, and gene expression during brain aging
Source: bioRxiv. 2026 Oct 21:2026.10.15.757659. Preprint. [Version 1] doi: 10.64898/2026.10.15.757659 (PMC13738379; doi:10.64898/2026.10.15.757659)
Supplement: Supplementary file 4 [file media-4.pdf]

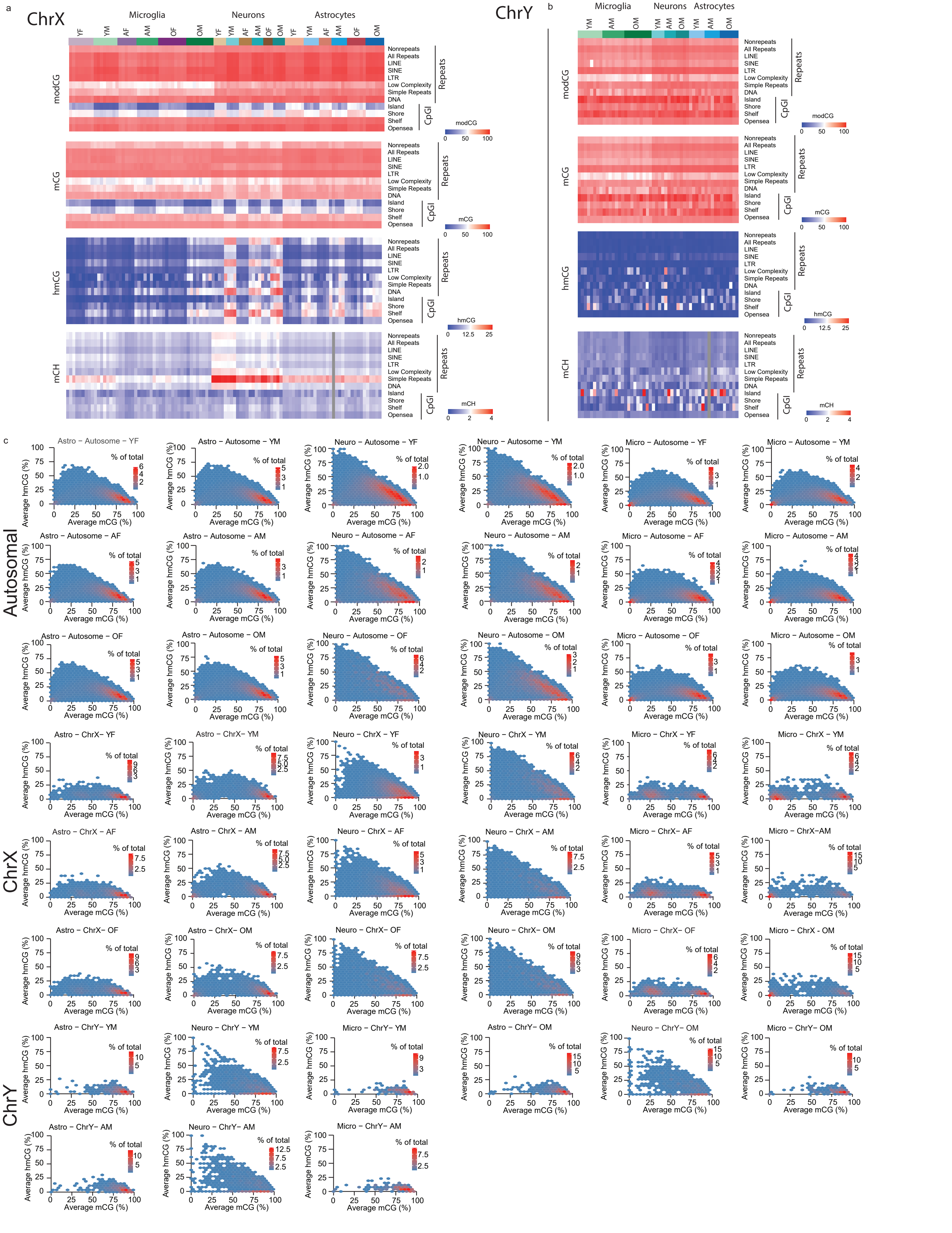

a
ChrX
Microglia
Neurons
Astrocytes
YF
YM
AF
AM
OF
OM
modCG
mCG
hmCG
mCH
Nonrepeats
All Repeats
LINE
SINE
LTR
Low Complexity
Simple Repeats
DNA
Island
Shore
Shelf
Opensea
Repeats
CpGI
b
ChrY
c
Autosomal
Astro – Autosome – YF
Astro – Autosome – YM
Neuro – Autosome – YF
Neuro – Autosome – YM
Micro – Autosome – YF
Micro – Autosome – YM
Astro – Autosome – AF
Astro – Autosome – AM
Neuro – Autosome – AF
Neuro – Autosome – AM
Micro – Autosome – AF
Micro – Autosome – AM
Astro – Autosome – OF
Astro – Autosome – OM
Neuro – Autosome – OF
Neuro – Autosome – OM
Micro – Autosome – OF
Micro – Autosome – OM
ChrX
Astro – ChrX– YF
Astro – ChrX– YM
Neuro – ChrX – YF
Neuro – ChrX – YM
Micro – ChrX – YF
Micro – ChrX – YM
Astro – ChrX – AF
Astro – ChrX – AM
Neuro – ChrX – AF
Neuro – ChrX – AM
Micro – ChrX– AF
Micro – ChrX–AM
Astro – ChrX– OF
Astro – ChrX– OM
Neuro – ChrX– OF
Neuro – ChrX– OM
Micro – ChrX– OF
Micro – ChrX - OM
ChrY
Astro – ChrY– YM
Neuro – ChrY – YM
Micro – ChrY– YM
Astro – ChrY– OM
Neuro – ChrY– OM
Micro – ChrY– OM
Astro – ChrY– AM
Neuro – ChrY– AM
Micro – ChrY– AM
% of total
Average hmCG (%)
Average mCG (%)
