# Supplementary material for: A new epigenetic framework for DNA methylation, hydroxymethylation, and gene expression during brain aging
Source: bioRxiv. 2026 Oct 21:2026.10.15.757659. Preprint. [Version 1] doi: 10.64898/2026.10.15.757659 (PMC13738379; doi:10.64898/2026.10.15.757659)
Supplement: Supplementary file 5 [file media-5.pdf]

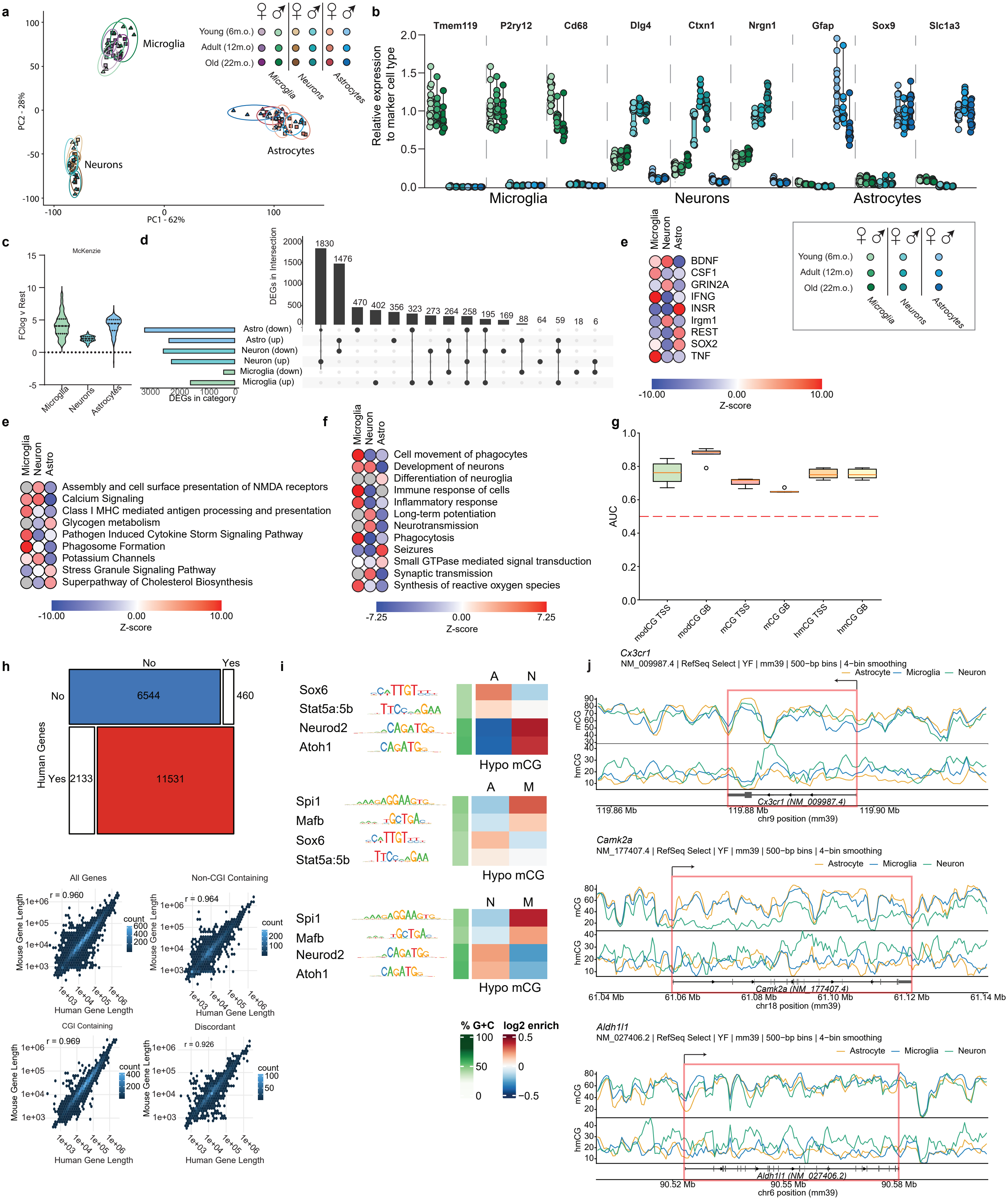
a
Microglia
Neurons
Astrocytes
PC1 - 62%
PC2 - 28%
Young (6m.o.)
Adult (12m.o)
Old (22m.o.)
b
Tmem119
P2ry12
Cd68
Dlg4
Ctxn1
Nrgn1
Gfap
Sox9
Slc1a3
Relative expression to marker cell type
Microglia
Neurons
Astrocytes
c
McKenzie
FClog v Rest
d
DEGs in Intersection
DEGs in category
Astro (down)
Astro (up)
Neuron (down)
Neuron (up)
Microglia (down)
Microglia (up)
1830
1476
470
402
356
323
273
264
258
195
169
88
64
59
18
6
e
Microglia
Neuron
Astro
BDNF
CSF1
GRIN2A
IFNG
INSR
Irgm1
REST
SOX2
TNF
Z-score
e
Assembly and cell surface presentation of NMDA receptors
Calcium Signaling
Class I MHC mediated antigen processing and presentation
Glycogen metabolism
Pathogen Induced Cytokine Storm Signaling Pathway
Phagosome Formation
Potassium Channels
Stress Granule Signaling Pathway
Superpathway of Cholesterol Biosynthesis
Z-score
f
Cell movement of phagocytes
Development of neurons
Differentiation of neuroglia
Immune response of cells
Inflammatory response
Long-term potentiation
Neurotransmission
Phagocytosis
Seizures
Small GTPase mediated signal transduction
Synaptic transmission
Synthesis of reactive oxygen species
Z-score
g
AUC
modCG TSS
modCG GB
mCG TSS
mCG GB
hmCG TSS
hmCG GB
h
No
Yes
Human Genes
6544
460
2133
11531
All Genes
Non-CGI Containing
CGI Containing
Discordant
r = 0.960
r = 0.964
r = 0.969
r = 0.926
Mouse Gene Length
Human Gene Length
count
i
Sox6
Stat5a:5b
Neurod2
Atoh1
Spi1
Mafb
A
N
M
Hypo mCG
% G+C
log2 enrich
j
Cx3cr1
NM_009987.4 | RefSeq Select | YF | mm39 | 500-bp bins | 4-bin smoothing
Astrocyte
Microglia
Neuron
mCG
hmCG
Cx3cr1 (NM_009987.4)
119.86 Mb
119.88 Mb
119.90 Mb
chr9 position (mm39)
Camk2a
NM_177407.4 | RefSeq Select | YF | mm39 | 500-bp bins | 4-bin smoothing
Camk2a (NM_177407.4)
61.04 Mb
61.06 Mb
61.08 Mb
61.10 Mb
61.12 Mb
61.14 Mb
chr18 position (mm39)
Aldh1l1
NM_027406.2 | RefSeq Select | YF | mm39 | 500-bp bins | 4-bin smoothing
Aldh1l1 (NM_027406.2)
90.52 Mb
90.55 Mb
90.58 Mb
chr6 position (mm39)
