# Supplementary material for: A new epigenetic framework for DNA methylation, hydroxymethylation, and gene expression during brain aging
Source: bioRxiv. 2026 Oct 21:2026.10.15.757659. Preprint. [Version 1] doi: 10.64898/2026.10.15.757659 (PMC13738379; doi:10.64898/2026.10.15.757659)
Supplement: Supplementary file 6 [file media-7.pdf]

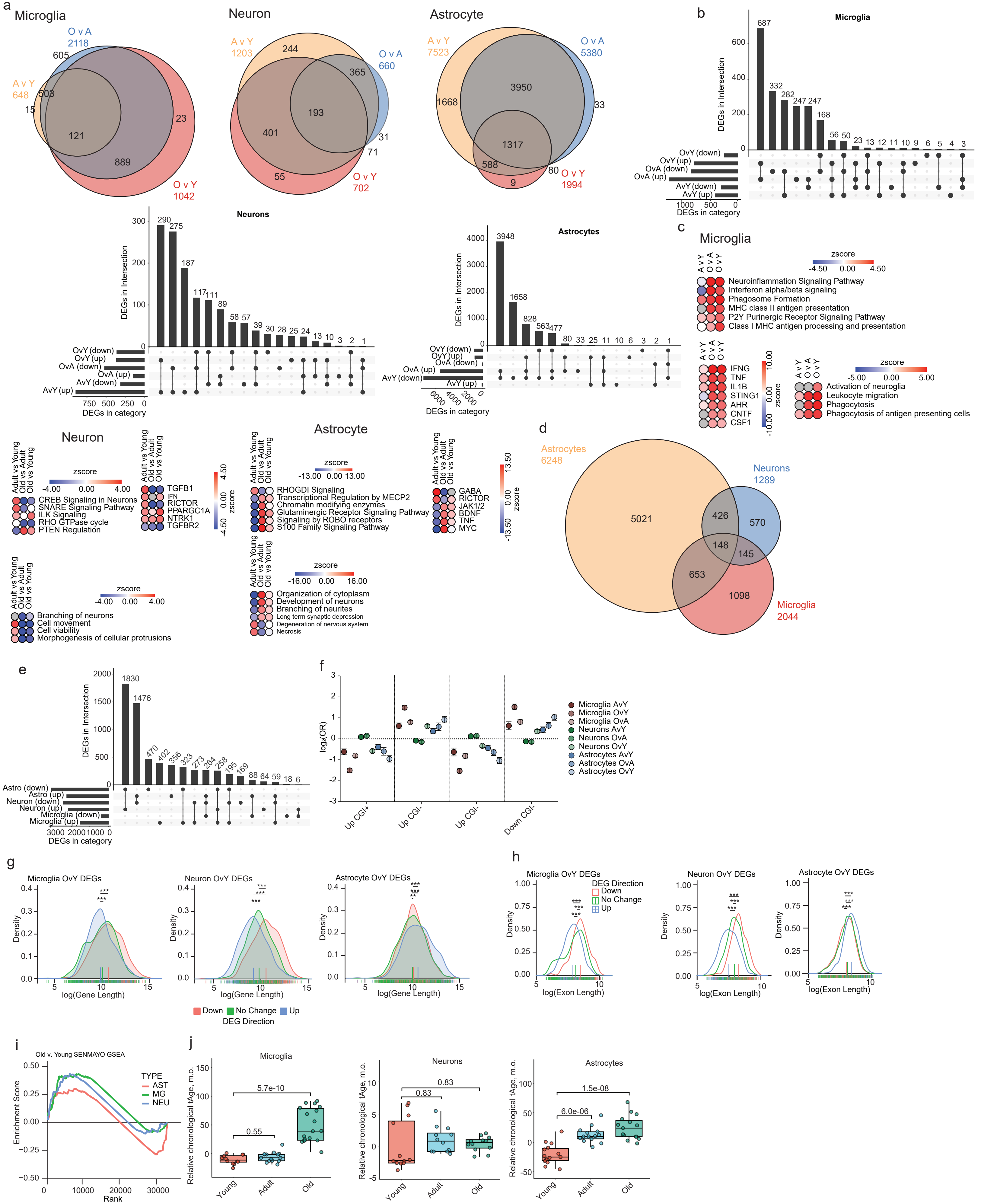

a
Microglia
O v A
2118
A v Y
648
605
503
15
121
23
889
O v Y
1042
Neuron
A v Y
1203
244
O v A
660
365
193
401
31
71
55
O v Y
702
Astrocyte
A v Y
7523
O v A
5380
3950
1668
33
1317
588
80
9
O v Y
1994
b
Microglia
DEGs in Intersection
687
332
282
247
247
168
56
50
23
13
12
11
10
9
6
5
4
3
OvY (down)
OvY (up)
OvA (down)
OvA (up)
AvY (down)
AvY (up)
1000
500
0
DEGs in category
Neurons
DEGs in Intersection
290
275
187
117
111
89
58
57
39
30
28
25
24
13
10
3
2
1
OvY (down)
OvY (up)
OvA (down)
OvA (up)
AvY (down)
AvY (up)
750
500
250
0
DEGs in category
Astrocytes
DEGs in Intersection
3948
1658
828
563
477
80
33
25
11
10
6
3
2
1
OvY (down)
OvY (up)
OvA (down)
OvA (up)
AvY (down)
AvY (up)
6000
4000
2000
0
DEGs in category
c
Microglia
A v Y
O v A
O v Y
zscore
-4.50
0.00
4.50
Neuroinflammation Signaling Pathway
Interferon alpha/beta signaling
Phagosome Formation
MHC class II antigen presentation
P2Y Purinergic Receptor Signaling Pathway
Class I MHC antigen processing and presentation
IFNG
TNF
IL1B
STING1
AHR
CNTF
CSF1
10.00
0.00
-10.00
zscore
-5.00
0.00
5.00
Activation of neuroglia
Leukocyte migration
Phagocytosis
Phagocytosis of antigen presenting cells
Neuron
Adult vs Young
Old vs Adult
Old vs Young
zscore
-4.00
0.00
4.00
CREB Signaling in Neurons
SNARE Signaling Pathway
ILK Signaling
RHO GTPase cycle
PTEN Regulation
TGFB1
IFN
RICTOR
PPARGC1A
NTRK1
TGFBR2
4.50
0.00
-4.50
zscore
Branching of neurons
Cell movement
Cell viability
Morphogenesis of cellular protrusions
Astrocyte
zscore
-13.00
0.00
13.00
RHOGDI Signaling
Transcriptional Regulation by MECP2
Chromatin modifying enzymes
Glutaminergic Receptor Signaling Pathway
Signaling by ROBO receptors
S100 Family Signaling Pathway
GABA
RICTOR
JAK1/2
BDNF
TNF
MYC
13.50
0.00
-13.50
zscore
-16.00
0.00
16.00
Organization of cytoplasm
Development of neurons
Branching of neurites
Long term synaptic depression
Degeneration of nervous system
Necrosis
d
Astrocytes
6248
Neurons
1289
5021
426
570
148
145
653
1098
Microglia
2044
e
DEGs in Intersection
1830
1476
470
402
356
323
273
264
258
195
169
88
64
59
18
6
Astro (down)
Astro (up)
Neuron (down)
Neuron (up)
Microglia (down)
Microglia (up)
3000
2000
1000
0
DEGs in category
f
log2(OR)
Up CGI+
Up CGI-
Up CGI-
Down CGI-
Microglia AvY
Microglia OvY
Microglia OvA
Neurons AvY
Neurons OvA
Neurons OvY
Astrocytes AvY
Astrocytes OvA
Astrocytes OvY
g
Microglia OvY DEGs
Neuron OvY DEGs
Astrocyte OvY DEGs
Density
log(Gene Length)
Down
No Change
Up
DEG Direction
h
Microglia OvY DEGs
Neuron OvY DEGs
Astrocyte OvY DEGs
DEG Direction
Down
No Change
Up
Density
log(Exon Length)
i
Old v. Young SENMAYO GSEA
Enrichment Score
Rank
TYPE
AST
MG
NEU
j
Microglia
Neurons
Astrocytes
Relative chronological tAge, m.o.
5.7e-10
0.55
0.83
0.83
1.5e-08
6.0e-06
Young
Adult
Old
