# Supplementary material for: A new epigenetic framework for DNA methylation, hydroxymethylation, and gene expression during brain aging
Source: bioRxiv. 2026 Oct 21:2026.10.15.757659. Preprint. [Version 1] doi: 10.64898/2026.10.15.757659 (PMC13738379; doi:10.64898/2026.10.15.757659)
Supplement: Supplementary file 7 [file media-8.pdf]

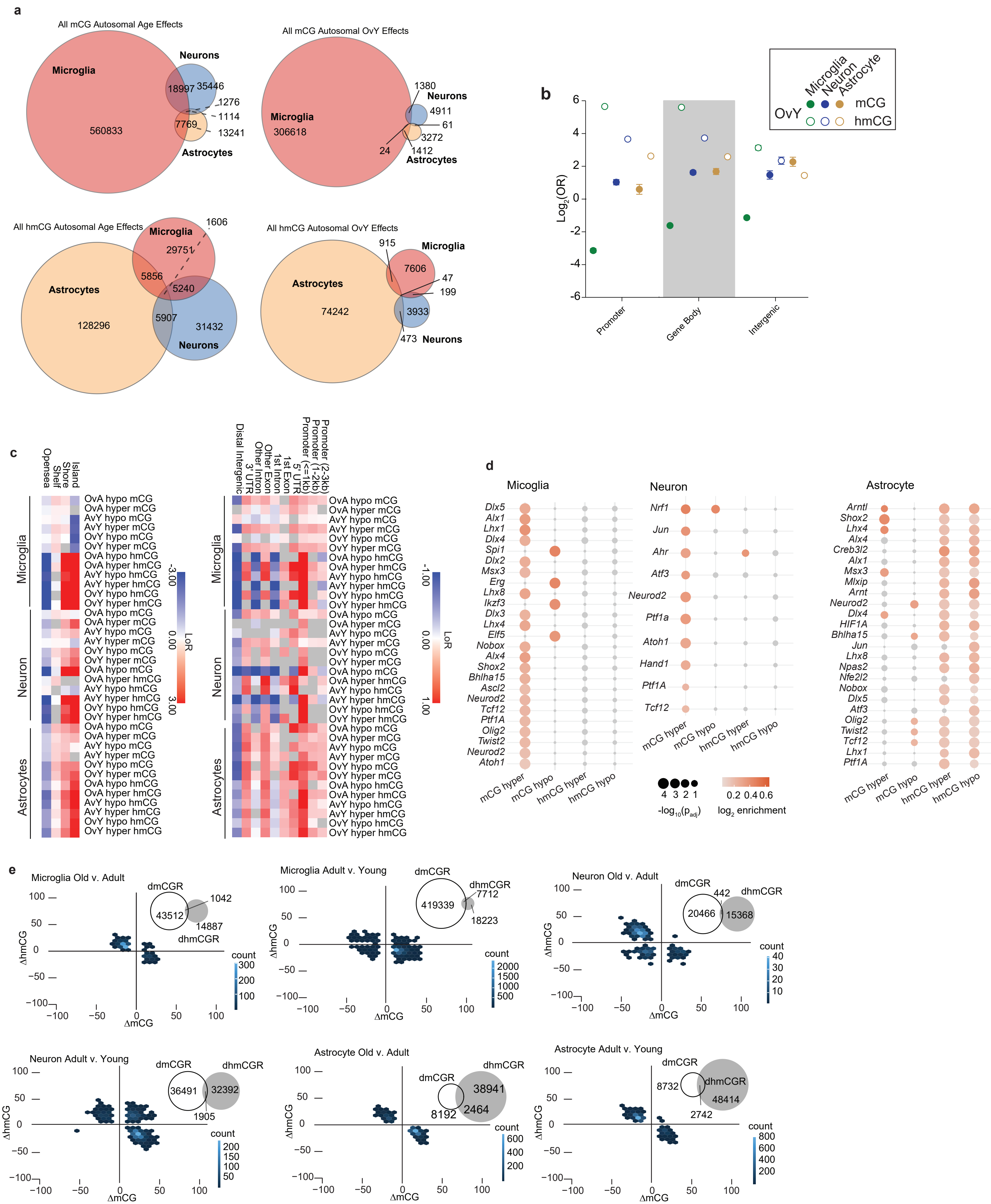
a
All mCG Autosomal Age Effects
Microglia
Neurons
Astrocytes
560833
18997
35446
1276
1114
7769
13241
All mCG Autosomal OvY Effects
Microglia
306618
1380
Neurons
4911
61
3272
24
1412
Astrocytes
All hmCG Autosomal Age Effects
Microglia
1606
29751
5856
5240
Astrocytes
128296
5907
31432
Neurons
All hmCG Autosomal OvY Effects
915
Microglia
7606
47
199
Astrocytes
74242
3933
473
Neurons
b
Microglia
Neuron
Astrocyte
OvY
mCG
hmCG
Log2(OR)
6
4
2
0
-2
-4
-6
Promoter
Gene Body
Intergenic
c
Opensea
Shelf
Shore
Island
Microglia
Neuron
Astrocytes
OvA hypo mCG
OvA hyper mCG
AvY hypo mCG
AvY hyper mCG
OvY hypo mCG
OvY hyper mCG
OvA hypo hmCG
OvA hyper hmCG
AvY hypo hmCG
AvY hyper hmCG
OvY hypo hmCG
OvY hyper hmCG
-3.00
0.00
3.00
LoR
Distal Intergenic
3' UTR
Other Intron
Other Exon
1st Intron
1st Exon
5' UTR
Promoter (<=1kb)
Promoter (1-2kb)
Promoter (2-3kb)
-1.00
0.00
1.00
LoR
d
Micoglia
Dlx5
Alx1
Lhx1
Dlx4
Spi1
Dlx2
Msx3
Erg
Lhx8
Ikzf3
Dlx3
Lhx4
Elf5
Nobox
Alx4
Shox2
Bhlha15
Ascl2
Neurod2
Tcf12
Ptf1A
Olig2
Twist2
Neurod2
Atoh1
mCG hyper
mCG hypo
hmCG hyper
hmCG hypo
Neuron
Nrf1
Jun
Ahr
Atf3
Neurod2
Ptf1a
Atoh1
Hand1
Ptf1A
Tcf12
mCG hyper
mCG hypo
hmCG hyper
hmCG hypo
4 3 2 1
-log10(padj)
0.2 0.4 0.6
log2 enrichment
Astrocyte
Arntl
Shox2
Lhx4
Alx4
Creb3l2
Alx1
Msx3
Mlxip
Arnt
Neurod2
Dlx4
HIF1A
Bhlha15
Jun
Lhx8
Npas2
Nfe2l2
Nobox
Dlx5
Atf3
Olig2
Twist2
Tcf12
Lhx1
Ptf1A
mCG hyper
mCG hypo
hmCG hyper
hmCG hypo
e
Microglia Old v. Adult
dmCGR
43512
1042
14887
dhmCGR
count
300
200
100
Microglia Adult v. Young
dmCGR
419339
dhmCGR
7712
18223
count
2000
1500
1000
500
Neuron Old v. Adult
dmCGR
20466
442
dhmCGR
15368
count
40
30
20
10
Neuron Adult v. Young
dmCGR
36491
dhmCGR
32392
1905
count
200
150
100
50
Astrocyte Old v. Adult
dmCGR
dhmCGR
38941
8192
2464
count
600
400
200
Astrocyte Adult v. Young
dmCGR
8732
dhmCGR
48414
2742
count
800
600
400
200
ΔhmCG
ΔmCG
100
50
0
−50
−100
