# Supplementary material for: A new epigenetic framework for DNA methylation, hydroxymethylation, and gene expression during brain aging
Source: bioRxiv. 2026 Oct 21:2026.10.15.757659. Preprint. [Version 1] doi: 10.64898/2026.10.15.757659 (PMC13738379; doi:10.64898/2026.10.15.757659)
Supplement: Supplementary file 8 [file media-9.pdf]

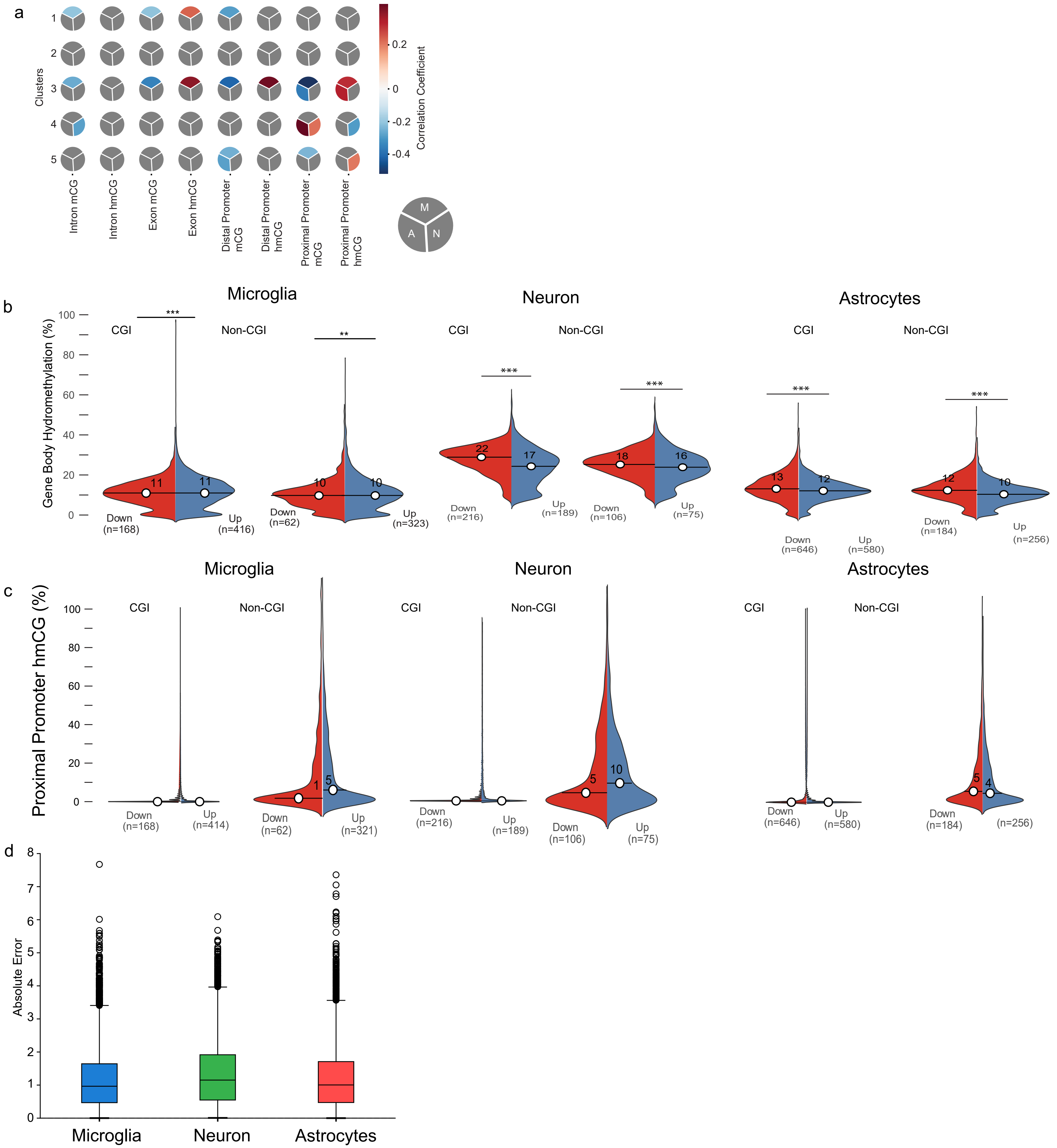

a
Clusters
1
2
3
4
5
Intron mCG
Intron hmCG
Exon mCG
Exon hmCG
Distal Promoter mCG
Distal Promoter hmCG
Proximal Promoter mCG
Proximal Promoter hmCG
Correlation Coefficient
0.2
0
-0.2
-0.4
M
A
N
b
Microglia
Neuron
Astrocytes
Gene Body Hydromethylation (%)
CGI
Non-CGI
Down (n=168)
Up (n=416)
Down (n=62)
Up (n=323)
Down (n=216)
Up (n=189)
Down (n=106)
Up (n=75)
Down (n=646)
Up (n=580)
Down (n=184)
Up (n=256)
c
Proximal Promoter hmCG (%)
Down (n=168)
Up (n=414)
Down (n=62)
Up (n=321)
Down (n=216)
Up (n=189)
Down (n=106)
Up (n=75)
Down (n=646)
Up (n=580)
Down (n=184)
(n=256)
d
Absolute Error
Microglia
Neuron
Astrocytes
